# Influence of *Pichia pastoris* X-33 produced in industrial residues on productive performance, egg quality, immunity, and intestinal morphometry in quails

**DOI:** 10.1038/s41598-019-51908-0

**Published:** 2019-10-25

**Authors:** Giana Carla Gaboardi, Débora Alves, Diego Gil de los Santos, Eduardo Xavier, Ana Paula Nunes, Paula Finger, Emili Griep, Victor Roll, Patrícia Oliveira, Arthur Silva, Ângela Moreira, Fabricio Conceição

**Affiliations:** 1 Centro de Desenvolvimento Tecnológico, Universidade Federal de Pelotas, Pelotas, Brazil; 2 Faculdade de Agronomia, Universidade Federal de Pelotas, Pelotas, Brazil; 3 Instituto Federal Sul-rio-grandense, Campus Pelotas, Pelotas, Brazil; 4 Faculdade de Medicina, Universidade Federal de Pelotas, Pelotas, Brazil; 5 Faculdade de Nutrição, Universidade Federal de Pelotas, Pelotas, Brazil

**Keywords:** Mucosal immunology, Applied microbiology

## Abstract

This study was conducted in quails to evaluate the probiotic potential of *Pichia pastoris* X-33, cultivated in parboiled rice effluent supplemented with biodiesel glycerol or in standard medium Yeast Extract–Peptone–Dextrose (YPD). Forty-days-old female quails were divided into three treatments: T1 (Control) received a basal diet without *P. pastoris*; T2 (*Pichia* Effluent) received a basal diet supplemented with *P. pastoris* grown in parboiled rice effluent and biodiesel glycerol, and T3 (*Pichia* YPD) received a basal diet supplemented with *P. pastoris* produced in YPD. The birds were vaccinated against Newcastle Disease (NDV), Avian Infectious Bronchitis (IBV), and Gumboro Disease on days 1 and 28. The following parameters were analyzed: performance, egg quality, humoral immune response to the vaccines, organ weight, and intestinal morphometry. *P. pastoris* grown in YPD increased egg weight (p < 0.05). The lowest liver weight on day 14 was obtained in *Pichia* Effluent, whereas both *P. pastoris* supplemented groups had the lowest duodenum weights on day 14. Besides that, livers and duodenums presented no morphological changes in any of the three treatments. Supplementation of *P. pastoris* modulated the immune system of the birds, increasing anti-IBV, anti-NDV, and anti-Gumboro antibodies levels compared to the Control (p < 0.05). In conclusion, quail’s immune response was improved by *Pichia pastoris* X-33, either it was grown in YPD or industrial residues, and the egg weight increased with *Pichia pastoris* X-33 grown in YPD, thereby demonstrating to be a promising probiotic for poultry.

## Introduction

For decades, it was a common practice to use antibiotics as growth promoters in animal production to increase food efficiency, promote growth, and improve the quality of products^1^. However, its indiscriminate use raised fear about the development of resistance mechanisms to antibiotics and the transfer of resistant bacteria from animals to humans, and for this reason, its use as growth promoters was first banned by the European Community^2,3^ and later by several other countries. Since then, a number of alternatives have been proposed and tested^4^ in a search for an ideal substitute that exerts the same effects: increased performance and immunomodulatory activity^5,6^.

In this sense, interest in probiotics for animal use has intensified, encouraging the exploration of new probiotic species as safe and low-cost alternatives to antibiotics^7,8^. Probiotics are living microorganisms that, when ingested in adequate doses, confer benefits to the host^9^. Animal diet supplementation with probiotics has positively modulated the immune response, decreased infection by enteropathogenic bacteria, increased food efficiency^10–12^, and improved egg quality in chickens^13–15^.

*Pichia pastoris* is a methylotrophic yeast that has been used as a recombinant protein expression system for over two decades; it is especially useful in the production of complex proteins that require post-translational modifications to fold and function correctly^16–18^. Some of special features of *P. pastoris* include high cell density production in simple and low-cost media and its GRAS (*Generally Recognized as Safe*) status, which ensures the safety of its application in therapeutic strategies and production of single-cell proteins^19^. Recently, explorations of its potential as a probiotic have revealed that *P. pastoris* exerts antimicrobial activity against *Salmonella* Typhimurium in mice^20^ and increases weight gain, improves feed conversion, and modulates the humoral immune response in broilers^21,22^. As a strategy to decrease the costs of probiotic production, studies has been conducted in this regard, showing the efficient growth of *P. pastoris* X-33 in agroindustrial waste. In this sense, *P. pastoris* can grow in parboiled rice effluent supplemented with glycerol byproduct of biodiesel, with large biomass production, high cellular viability, reduction of chemical oxygen demand (COD), and removal of nitrogen and phosphorus to levels required by environmental inspection agencies^23^, making it possible to treat the effluent and add value as a culture medium. These results suggest that *P. pastoris* may be produced at a low cost for beneficial applications as a probiotic in poultry.

Quail farming is a sector of poultry currently expanding in Brazil, where it is designed for the production of meat and eggs. In the last decades, quail farming was no longer considered a subsistence practice and began to occupy a position of highly-qualified activity with promising results for investors^24,25^. Among the attractive characteristics of quails are fast growth, early sexual maturity, early posture, short incubation period, high productivity, persistence in egg production, housing of large populations in small spaces and low cost of housing^25–27^. Some previous studies in quails have already indicated the beneficial effects of probiotic administration on performance, egg quality, blood biochemical parameters, intestinal morphology, immunomodulation, and protection against toxins^28–30^.

Considering the reported benefits of *Pichia pastoris* as a probiotic and the lack of data about its effect in quails, this study was conducted to evaluate how *Pichia pastoris* X-33, cultivated in either standard culture medium or industrial effluent, affects quail performance, egg quality, intestinal morphology, and immune response modulation.

## Results

### *Pichia pastoris* X-33 cell viability and stability

In both Yeast Extract–Peptone–Dextrose (YPD) and alternative medium (effluent supplemented with biodiesel glycerol), 10^8^ CFU.mL^−1^ was obtained. *P. pastoris* cell pellets were kept refrigerated at 4 °C, resuspended in 0.9% saline solution throughout the experiment (84 days). During this period, cell viability decreased in the last month only, by 0.5 log. mL^−1^. In the diets, yeast viability was maintained without a decrease in the concentration of viable cells, probably because fresh yeast was mixed into the diet every week.

### Performance

The performance variables analyzed during the experiment are shown in Table 1. The initial body weight of the birds was statistically equivalent in all groups (p > 0.05). The body weight was statistically higher in *Pichia* Effluent group in relation to *Pichia* YPD group on day 84, with a difference of 26 g. Numerically, *Pichia* Effluent group had the highest weight values after day 28. These results also reflected in the weight gain, which showed the same pattern, with a significant difference between the *Pichia* Effluent and *Pichia* YPD groups. Feed intake was maintained throughout the experimental period at approximately 42 g/bird/day. No group differed significantly in egg production (%), although egg production increased numerically during the analyzed period in the *Pichia* groups. On day 28, the control group obtained the worst feed conversion, compared to both of the groups supplemented with *P. pastoris*. After day 28, this parameter improved numerically but non-significantly in all the groups.

**Table 1:** Productive performance of quails fed diets containing *Pichia pastoris* X-33 grown in parboiled rice effluent supplemented with biodiesel glycerol or in YPD medium.

	Time	Treatments			SEM	*P*-value		
		Control	*Pichia* Effluent	*Pichia* YPD		Treatment	Time	Treat*Time
Body weight (g)	0 d	305.30	301.27	305.26	2.600	0.0322	<0.0001	0.9571
	0–28 d	363.91	370.05	359.20				
	28–56 d	389.06	397.58	384.99				
	56–84 d	404.93^ab^	415.98^a^	389.65^b^				
Body weight gain (g)	0–28 d	58.61	68.78	53.94		0.0110	<0.0001	0.9799
	28–56 d	83.77	96.31	79.74	2.501			
	56–84 d	99.63^ab^	114.70^a^	84.40^b^				
Feed intake (g/d)	0–28 d	42.52	42.51	41.59	0.334	0.5488	0.8701	0.8730
	28–56 d	42.66	42.53	41.89				
	56–84 d	41.89	43.43	42.47				
	**Average**	**42.35**	**42.82**	**41.98**				
Egg production (%)	0–28 d	78.53	79.23	85.28	1.242	0.9047	0.0010	0.2772
	28–56 d	93.23	86.48	85.37				
	56–84 d	91.21	93.54	91.44				
	**Average**	**87.66**	**86.42**	**87.36**				
Egg mass (g/bird/d)	0–28 d	11.30	11.22	11.68	0.432	0.5881	0.2711	0.794
	28–56 d	13.98	12.70	12.25				
	56–84 d	13.87	13.64	13.33				
	**Average**	**13.05**	**12.52**	**12.42**				
Feed conversion/Egg mass (g/g)	0–28 d	5.19	4.30	3.81	0.153	0.7984	0.0019	0.2127
	28–56 d	3.11	3.75	3.62				
	56–84 d	3.07	3.18	3.25				
	**Average**	**3.79**	**3.74**	**3.56**				

Different superscript letters in the same row indicate significant difference between averages (*P* < 0.05) by Tukey’s test; SEM = Standard error of the mean. Control = basal diet; *Pichia* Effluent = basal diet + *P. pastoris* X-33 grown in parboiled rice effluent supplemented with biodiesel glycerol; *Pichia* YPD = basal diet + *P. pastoris* X-33 grown in YPD medium.

### Egg quality

Internal egg quality variables are described in Table 2. The average egg weight in the *Pichia* YPD group was significantly higher than in control group (p < 0.05), with an increase of 1 g in egg weight between days 28 and 56, and an increase of 1.7 g between days 56 and 84, in the *Pichia* YPD group compared to the control group. On day 28, the highest yolk percentages were obtained in the control and *Pichia* YPD groups, which did not differ from each other, but both were significantly higher than the *Pichia* Effluent group (p < 0.05). After day 28, there was no significant variation in the yolk percentages between the groups. The other internal egg quality variables (yolk weight, albumen percentage, albumen weight) and yolk color showed no variation or difference between groups at any point during the experiment, except for the difference in the parameter a* (redness) in *Pichia* YPD and control groups on day 84 (Table 3). No significant difference was found in the Haugh Unit scores, but a gradual increase occurred in all treatments from day 28 onwards.

**Table 2:** Internal egg quality parameters of quails fed diets containing *Pichia pastoris* X-33 grown in parboiled rice effluent supplemented with biodiesel glycerol or in YPD medium.

	Time	Treatments			SEM	*P*-value		
		Control	*Pichia* Effluent	*Pichia* YPD		Treatment	Time	Treat*Time
Egg weight (g)	0–28 d	14.24	14.52	14.20	0.108	0.0254	0.0664	0.8204
	28–56 d	14.37^b^	15.11^ab^	15.35^a^				
	56–84 d	14.24^b^	15.25^ab^	15.95^a^				
	**Average**	**14.04**	**14.96**	**15.16**				
Yolk weight (g)	0–28 d	4.24	4.13	4.29	0.037	0.9417	0.0005	0.6902
	28–56 d	4.57	4.50	4.38				
	56–84 d	4.51	4.57	4.62				
	**Average**	**4.44**	**4.40**	**4.43**				
Yolk ratio (%)	0–28 d	29.02^a^	26.88^b^	30.20^a^	0.247	0.0137	0.0237	0.1942
	28–56 d	29.38	29.78	30.62				
	56–84 d	29.94	29.98	30.87				
	**Average**	**29.45**	**28.88**	**30.57**				
Albumen weight (g)	0–28 d	7.61	7.59	7.27	0.069	0.1248	0.1012	0.9664
	28–56 d	7.78	8.03	7.61				
	56–84 d	7.78	7.98	7.66				
	**Average**	**7.72**	**7.86**	**7.51**				
Albumen ratio (%)	0–28 d	51.85	49.49	51.26	0.466	0.4968	0.2143	0.2740
	28–56 d	51.44	53.08	52.98				
	56–84 d	48.50	52.33	51.19				
	**Average**	**50.59**	**51.63**	**51.81**				
Haugh Unit	0–28 d	92.26	92.00	92.71	0.365	0.8224	0.0532	0.6890
	28–56 d	92.50	92.29	92.41				
	56–84 d	94.01	95.69	93.22				
	**Average**	**92.92**	**93.33**	**92.78**				

Different superscript letters in the same row indicate significant difference between averages (*P* < 0.05) by Tukey’s test; SEM = Standard error of the mean. Control = basal diet; *Pichia* Effluent = basal diet + *P. pastoris* X-33 grown in parboiled rice effluent supplemented with biodiesel glycerol; *Pichia* YPD = basal diet + *P. pastoris* X-33 grown in YPD medium.

**Table 3:** Egg yolk color parameters of quails fed diets containing *Pichia pastoris* X-33 grown in parboiled rice effluent supplemented with biodiesel glycerol or in YPD medium.

	Time	Treatments			SEM	*P*-value		
		Control	*Pichia* Effluent	*Pichia* YPD		Treatment	Time	Treat*Time
Yolk color (DSM^®^ color fan)	0–28 d	3.80	3.91	4.10	0.037	0.7723	<0.0001	0.1197
	28–56 d	4.16	4.31	4.23				
	56–84 d	3.91	3.73	3.71				
	**Average**	**3.95**	**3.98**	**4.01**				
Lightness (L*)	0–28 d	56.81	58.82	57.15	0.297	0.3562	0.0001	0.4229
	28–56 d	59.62	59.86	59.44				
	56–84 d	56.18	57.89	58.05				
	**Average**	**57.54**	**58.86**	**58.21**				
Redness (a*)	0–28 d	-5.47	-5.58	-5.44	0.059	0.0366	<0.0001	0.1539
	28–56 d	-4.92	-4.76	-5.00				
	56–84 d	-5.68^b^	-5.98^ab^	-6.21^a^				
	**Average**	**-5.36**	**-5.44**	**-5.55**				
Yellowness (b*)	0–28 d	35.51	36.37	35.18	0.308	0.1573	<0.0001	0.9582
	28–56 d	39.68	40.57	38.59				
	56–84 d	36.35	37.00	36.26				
	**Average**	**37.18**	**37.98**	**36.68**				

Different superscript letters in the same row indicate significant difference between averages (*P* < 0.05) by Tukey’s test; SEM = Standard error of the mean. Control = basal diet; *Pichia* Effluent = basal diet + *P. pastoris* X-33 grown in parboiled rice effluent supplemented with biodiesel glycerol; *Pichia* YPD = basal diet + *P. pastoris* X-33 grown in YPD medium.

The external egg quality measurements can be observed in Table 4. There was no significant difference in specific gravity between the treatments; however, the *Pichia* Effluent and *Pichia* YPD groups maintained the values throughout the experiment, while the control treatment had a progressive decrease in the specific gravity throughout the experiment. The eggshell weight was equal between treatments on all the days analyzed, with increase of 0.04 g in the final eggshell weights, in relation to initial weight in all treatments. On day 56, the highest eggshell percentage was observed in the *Pichia* YPD treatment, but it was not statistically different from the *Pichia* Effluent treatment and control group (p > 0.05). The eggshell thickness remained unchanged, without variations between the periods analyzed and between treatments.

**Table 4:** External egg quality parameters of quails fed diets containing *Pichia pastoris* X-33 grown in parboiled rice effluent supplemented with biodiesel glycerol or in YPD medium.

	Time	Treatments			SEM	*P*-value		
		Control	*Pichia* Effluent	*Pichia* YPD		Treatment	Time	Treat*Time
Specific gravity	0–28 d	1074.80	1075.13	1074.80	3.729	0.4097	0.6756	0.5044
	28–56 d	1069.50	1069.13	1070.80				
	56–84 d	1046.00	1077.69	1076.50				
	**Average**	**1063.43**	**1073.98**	**1074.03**				
Eggshell weight (g)	0–28 d	1.15	1.14	1.13	0.010	0.5096	0.1717	0.9905
	28–56 d	1.16	1.12	1.13				
	56–84 d	1.19	1.18	1.16				
	**Average**	**1.17**	**1.15**	**1.14**				
Eggshell ratio (%)	0–28 d	7.88	7.85	7.92	0.048	0.3178	0.0567	0.5900
	28–56 d	7.56	7.41	7.86				
	56–84 d	7.83	7.74	7.77				
	**Average**	**7.75**	**7.67**	**7.85**				
Eggshell thickness (mm)	0–28 d	0.290	0.287	0.291	0.002	0.7663	0.0511	0.9042
	28–56 d	0.298	0.298	0.307				
	56–84 d	0.299	0.297	0.296				
	**Average**	**0.296**	**0.294**	**0.298**				

Different superscript letters in the same row indicate significant difference between averages (*P* < 0.05) by Tukey’s test; SEM = Standard error of the mean. Control = basal diet; *Pichia* Effluent = basal diet + *P. pastoris* X-33 grown in parboiled rice effluent supplemented with biodiesel glycerol; *Pichia* YPD = basal diet + *P. pastoris* X-33 grown in YPD medium.

### Humoral immune response

**Figure 1:**
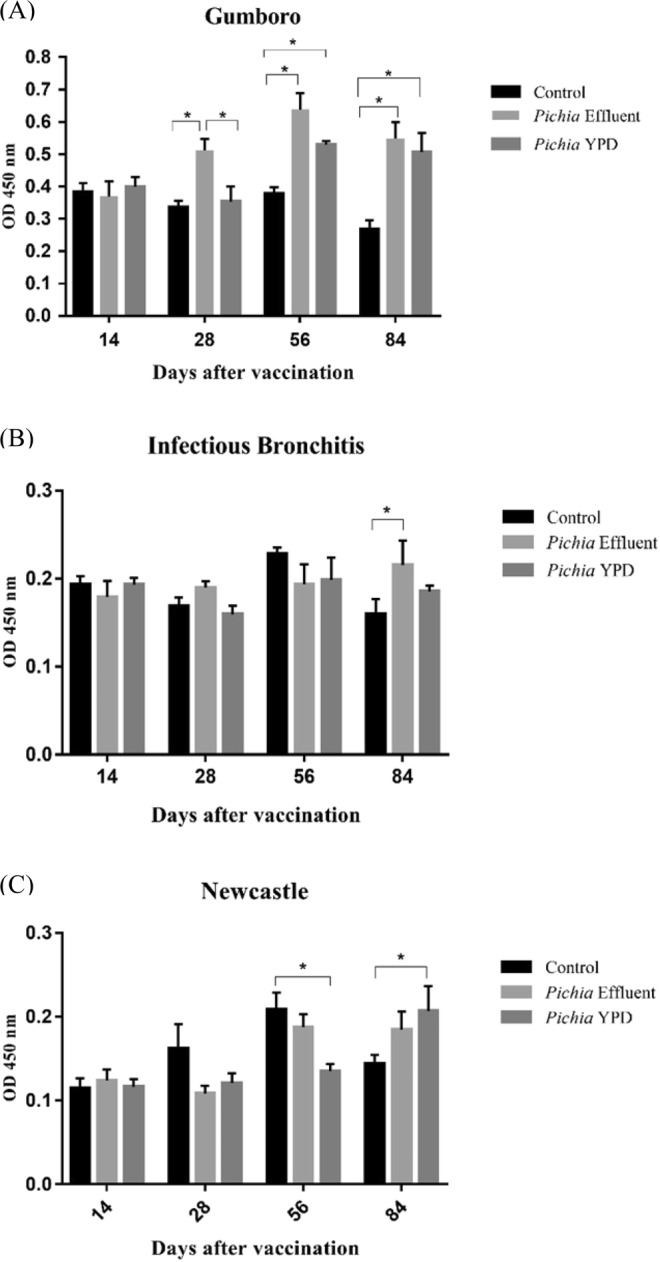
Evaluation by ELISA of antibodies anti - Gumboro disease (**A**), anti - Avian Infectious Bronchitis (**B**) and anti - Newcastle (**C**) of quails vaccinated against these diseases and fed diets containing *P. pastoris* X-33 grown in parboiled rice effluent supplemented with biodiesel glycerol or in YPD medium. Control = basal diet; *Pichia* Effluent = basal diet + *P. pastoris* X-33 grown in parboiled rice effluent supplemented with biodiesel glycerol; *Pichia* YPD = basal diet + *P. pastoris* X-33 grown in YPD medium. *Significant difference between averages (*P* < 0.05) by Tukey’s test.

Blood samples from days 14, 28, 56, and 84 were collected and tested by indirect ELISA, using as antigens the monovalent vaccines against Gumboro Disease, Infectious Bronchitis, and Newcastle Disease. The best antibody response was observed against Gumboro Disease (Fig. 1A), with higher absorbances at 56 and 84 days post-vaccination in both the *Pichia* Effluent and *Pichia* YPD groups compared to the control (p < 0.05). On day 84, the levels of anti-Gumboro antibodies in *P. pastoris* supplemented groups were approximately twice higher than in the control group. On day 28, the *Pichia* Effluent group had higher levels of anti-Gumboro antibodies than the other groups. The production of anti-Newcastle antibodies was greater in the *Pichia* YPD group than in the control group on day 84 (Fig. 1C), whereas in the same period, the *Pichia* Effluent group had a higher response against the Infectious Bronchitis virus (Fig. 1B) compared to the control group (p = 0.0657). The anti-NDV antibodies titers obtained by the hemagglutination inhibition (HI) test were also higher on day 84 in the *Pichia* YPD group compared to the control group (p < 0.05), with a 1.8-fold increase in the title (Table 5). The control group had the lowest titer of anti-NDV antibodies in this period. There was no difference in anti-NDV antibodies titers between the *Pichia* Effluent and *Pichia* YPD groups in any of the analyzed periods.

**Table 5:** Anti-NDV antibodies titer (log_2_), obtained by hemagglutination inhibition test (HI), of quails vaccinated against Newcastle Disease (NDV) and fed diets containing *P. pastoris* X-33 grown in parboiled rice effluent supplemented with biodiesel glycerol or in YPD medium.

Days after vaccination	Control	*Pichia* Effluent	*Pichia* YPD	SEM	*P*-value
14 d	2.75	2.75	2.5	0.083	0.9499
28 d	2.0	2.75	3.0	0.300	0.5785
56 d	5.0	4.25	4.5	0.220	0.4355
84 d	4.25^b^	6.0^ab^	7.5^a^	0.939	0.0097

Different superscript letters in the same row indicate significant difference between averages (*P* < 0.05) by Tukey’s test; SEM = Standard error of the mean. Control = basal diet; *Pichia* Effluent = basal diet + *P. pastoris* X-33 grown in parboiled rice effluent supplemented with biodiesel glycerol; *Pichia* YPD = basal diet + *P. pastoris* X-33 grown in YPD.

### Relative organ weights

Table 6 shows the relative weight of internal organs (g/100 g body weight) of quails of the *Pichia* Effluent, *Pichia* YPD, and control groups. The treatments had no effect on the relative weight of the heart, which was maintained throughout the experiment. In addition, there was no significant difference between treatments for the relative weight of lymphoid organs (spleen, bursa of Fabricius, and cecal tonsils) in any of the analyzed periods. The relative weight of the liver was lower in the *Pichia* Effluent group compared to the control group on day 14 (p < 0.05). Supplementation with *P. pastoris*, produced in both culture media tested, significantly reduced the weight of the duodenum on days 14 and 84 when compared to the control group.

**Table 6:** Relative weight of organs (g/100 g body weight) of quails fed diets containing *Pichia pastoris* X-33 grown in parboiled rice effluent supplemented with biodiesel glycerol or in YPD medium.

Organ	Time	Treatments			SEM	*P*-value
		Control	*Pichia* Effluent	*Pichia* YPD		
Heart	14 d	0.80	0.83	0.78	0.015	0.8506
	28 d	0.70	0.72	0.83	0.038	0.1467
	56 d	0.74	0.77	0.80	0.019	0.5986
	84 d	0.65	0.75	0.76	0.035	0.4898
Liver	14 d	2.55^a^	1.94^b^	2.13^ab^	0.178	0.0431
	28 d	2.02	1.75	1.87	0.079	0.3935
	56 d	1.96	2.12	2.00	0.047	0.7308
	84 d	1.59	1.76	1.41	0.102	0.3911
Spleen	14 d	0.08	0.06	0.08	0.008	0.3107
	28 d	0.05	0.05	0.04	0.004	0.5963
	56 d	0.07	0.09	0.05	0.012	0.2690
	84 d	0.06	0.06	0.04	0.007	0.4335
Bursa of Fabricius	14 d	0.05	0.08	0.06	0.007	0.8043
	28 d	0.04	0.03	0.03	0.004	0.5121
	56 d	0.01	0.02	0.01	0.002	0.2955
	84 d	0.01	0.03	0.04	0.009	0.4206
Duodenum	14 d	1.49^a^	1.04^b^	1.10^b^	0.143	0.0295
	28 d	0.99	1.01	1.01	0.005	0.9888
	56 d	1.00	0.90	1.00	0.033	0.6906
	84 d	0.87^a^	0.86^ab^	0.72^b^	0.051	0.0489
Ceca	14 d	0.65	0.79	0.63	0.051	0.7005
	28 d	0.43	0.49	0.61	0.052	0.3506
	56 d	0.55	0.49	0.68	0.055	0.3206
	84 d	0.57	0.51	0.48	0.026	0.7299

Different superscript letters in the same row indicate significant difference between averages (*P* < 0.05) by Tukey’s test; SEM = Standard error of the mean. Control = basal diet; *Pichia* Effluent = basal diet + *P. pastoris* X-33 grown in parboiled rice effluent supplemented with biodiesel glycerol; *Pichia* YPD = basal diet + *P. pastoris* X-33 grown in YPD medium.

### Intestinal morphometry and safety

The results of intestinal morphometry are shown in Table 7. Supplementation with *P. pastoris* X-33 grown in standard or alternative medium did not interfere significantly in the villi height nor crypt depth (p > 0.05). An increase in average values of villi height and crypt depth was observed in all groups throughout the experiment. The analysis of the liver and duodenum samples did not reveal any lesions in any of the groups tested.

**Table 7:** Morphometric analysis of villus height and crypt depth in duodenum of quails fed diets containing *Pichia pastoris* X-33 grown in parboiled rice effluent supplemented with biodiesel glycerol or in YPD medium.

	Time	Treatments			SEM	*P*-value
		Control	*Pichia* Effluent	*Pichia* YPD		
Villus height (µm)	14 d	982	1,026	1,008	8.56	0.0691
	28 d	997	1,040	1,018	7.80	0.1990
	56 d	1,013	1,034	1,036	9.50	0.4454
	84 d	1,052	1,065	1,069	9.95	0.8771
Crypt depth (µm)	14 d	158	147	125	1.58	0.5547
	28 d	152	190	157	1.73	0.4891
	56 d	176	192	161	1.47	0.7159
	84 d	191	194	166	2.06	0.9330

Different superscript letters in the same row indicate significant difference between averages (*P* < 0.05) by Tukey’s test; SEM = Standard error of the mean. Control = basal diet; *Pichia* Effluent = basal diet + *P. pastoris* X-33 grown in parboiled rice effluent supplemented with biodiesel glycerol; *Pichia* YPD = basal diet + *P. pastoris* X-33 grown in YPD medium.

## Discussion

One of the requirements for a microorganism to be used as probiotic is the ability to preserve its viability for long periods of storage. In this study, *P. pastoris* remained viable for approximately three months without a significant decrease in concentration or impairment of viability. This finding concurs with França *et al*.^20^, which had already reported at least two months viability of *P. pastoris* in the diet of mice.

*P. pastoris* X-33 cultivated in parboiling rice effluent supplemented with biodiesel glycerol was tested for its probiotic properties in mixed-breed quails and was found to confer a positive effect mainly on the immunity of the animals. Additionally, *P. pastoris* did not cause harmful effects on performance nor egg quality. The use of probiotics has already been evaluated in other types of animal production and beneficial effects have been demonstrated in pigs^12,31,32^, ruminants^33–37^, and poultry. In poultry farming, the main probiotics effects observed are the improvement on broiler performance and antimicrobial activity against intestinal pathogens such as *Salmonella* spp., *Campylobacter* spp., *Escherichia coli*, and *Clostridium perfringens*^38–43^. In quails, the administration of a single probiotic species or a probiotic consortium has been demonstrated to promote weight gain, improve blood biochemical parameters such as decrease on triglycerides and cholesterol levels^30^, protect against aflatoxins present in the diet, and modulate the immune system^28,29^.

Supplementation of the diet of quails with *P. pastoris* did not exert significant effects on the productive parameters (weight, weight gain, and feed conversion) compared to the control group, although numerically, the highest values of weight and weight gain were found in the *Pichia* Effluent group. Seifi and collaborators^30^ tested a commercial probiotic in one-day-old quails and concluded that early administration of probiotics was an essential factor to be possible to observe the probiotic’s positive effects in the animals. This may be because newborn chicks do not have yet a fully-formed microbiota and may be contaminated with pathogenic species from the non-sterile environment of incubators. Thus, the inclusion of probiotics in early life may protect against opportunistic pathogens by colonizing the gastrointestinal tract with beneficial species and thereby, preventing the attachment of intestinal pathogens^42^. In this study, *P. pastoris* was first administered when the quails were already 40 days old, which may have inhibited the observation of more pronounced effects of yeast activity.

Additionally, the potentiation of probiotic effect seems to be linked to two factors: inappropriate housing conditions and the presence of health challenges^44^. The work of Jin *et al*.^45^ supports this theory, observing that broilers subjected to an average temperature of 30 °C and 95% relative humidity showed improvement in feed efficiency when fed with a diet supplemented with *Lactobacillus acidophilus* or with a mixture of 12 *Lactobacillus* strains. Besides that, studies have reported a positive influence of probiotics on the performance and intestinal morphology of chickens that were challenged with different intestinal pathogens^46–48^. Throughout the present experiment, the animals were kept under conditions of thermal comfort in a properly sanitized room, with water *ad libitum*, and they were not subjected to any type of stress or challenge with pathogenic species, which may have masked the beneficial effects of *P. pastoris*.

Quails that were fed with a basal diet containing *P. pastoris* X-33 grown in YPD had an increase on egg weight by 7% (1.0 g) at 56 days and 12% (1.7 g) at 84 days, compared to the control (p < 0.05). These results are similar to those obtained in laying hens with the dietary supplementation of *Bacillus subtilis*^49^ and *Pediococcus acidilactici*^50^, and higher than those observed in chickens receiving *Bacillus licheniformis*^51^. The possibility of increment in the egg weight is desirable from an economic viewpoint since it enhances the acceptability by the consumer.

Eggshell quality was not influenced by yeast supplementation. Although no statistical difference had been obtained between the groups in the specific gravity, an indicator of eggshell quality, the control group showed a decrease in this parameter in the end of the experiment, while the groups receiving *P. pastoris* maintained the values throughout the period. The action of probiotics in the intestinal tract creates a more favorable environment by decreasing luminal pH, increasing the solubility of nutrients such as calcium, nitrogen, and phosphorus and thus, improves their absorption^49,50,52,53^. The inclusion of *Saccharomyces cerevisiae* cell lysate in the diet of laying hens increased egg weight, egg production, and improved feed conversion^54^. The authors also attributed these improvements to the characteristics of yeast cell wall components, which bind to the cell surface of some bacteria and prevent colonization, reducing the load of bacterial pathogens in the gut, which allows to the nutrients to be assimilated efficiently and targeted to egg production^55,56^. The yolk percentage did not increase with *P. pastoris* supplementation, reinforcing a pattern that had already been observed in studies using other probiotic species in laying hens^14,50^ and in quails that received dry bakery yeast^57^.

Yolk color was not affected either by the inclusion of *P. pastoris* in the diets. Yolk coloration is influenced mostly by the deposition of dietary carotenoids, mainly from corn, in the egg yolk^58^. Since the corn percentage in the diet did not vary among the treatments, this result was expected. However, Mikulski *et al*.^50^ obtained a higher score in yolk color in probiotic-treated groups than in the control and proposed that probiotics may improve carotenoid absorption and deposition in the yolk. Even so, our result is considered positive, since it suggests that the yeast can be used to improve the immune status of the birds without affecting the yolk color, which is an important factor for consumer acceptance of the product.

The Haugh Unit is generally used to evaluate albumen quality, which in turn is closely linked to egg freshness. Albumen quality is most influenced by factors as lineage, age of the animals, and egg storage time^58^, whereas nutritional characteristics seem do not influence in HU^59^, as seen in the present study with *P. pastoris* supplementation.

The results of this work confirmed data reported in the literature that probiotics increment or modulate the immune response^60,61^. The analysis showed that supplementation of the quail diet with *P. pastoris* enhanced the anti-NDV, anti-IBV, and anti-Gumboro immune responses. Indirect ELISA and HI showed the same trends on quantification of anti-NDV antibodies. In both tests, sera on day 84 exhibited an increase of up to 76% in the anti-NDV response in the *Pichia* YPD group compared to the control. Kasmani *et al*.^28^ reported an increase of 82% in the anti-NDV titer when *Brevibacillus laterosporus* was included in the quail diet, and they recently reported that quails fed with a diet containing a commercial probiotic consortium had an anti-NDV titer twice higher than the control group^29^. In relation to IBV, treatments with *P. pastoris* minimally stimulated the production of specific antibodies, with an increase of 35% in the *Pichia* Effluent group relative to the control group at 84 days. This result reflects observations in broilers, in which anti-IBV titers in groups treated with probiotics did not differ significantly from the other groups^62,63^, although the probiotic *Lactobacillus casei* caused immunomodulatory activity in the anti-IBV response of laying hens^64^.

Production of anti-Gumboro antibodies was higher in the *Pichia* Effluent group (p < 0.05), with an increase of 52% at 28 days, 69% at 56 days, and 104% at 84 days relative to the control group. Similarly, Gil de los Santos *et al*.^22^ confirmed the immunomodulatory activity of *P. pastoris* produced in this same effluent, which generated in broilers, a higher anti-Gumboro antibodies titer than the control at 28 days. The same author had already demonstrated the potential of *P. pastoris* X-33 as a bioremediator microorganism, finding that the yeast grown in parboiled rice effluent supplemented with 15 g.L^−1^ of biodiesel glycerol promoted reductions in COD - chemical oxygen demand (55%), phosphorus (52%), and nitrogen (45%)^23^. Recently, the cultivation of the probiotic *Saccharomyces boulardii* in this effluent reduced COD, nitrogen and phosphorus concentrations, reinforcing the possibility of producing probiotic yeasts in effluent and simultaneously reducing the environmental parameters^65^. These combined results bring a new approach to *P. pastoris*, which has been used for decades as a heterologous system for protein expression^16^.

The relative weights of the heart, spleen, bursa of Fabricius, and cecal tonsils did not vary with the addition of *P. pastoris* X-33, in concurrence with observations of of *Lactobacillus* spp. consumption by broilers^66,67^ and quails^29^. The two groups fed with *P. pastoris* had lower relative duodenum weights than the control group on day 14 (p < 0.05). The literature reports controversial findings on the liver weight; some studies indicate that probiotics increase liver weight^45,68^ while others do not demonstrate the influence of probiotics^29,67,69^. Contrary to these reports, the group supplemented with *P. pastoris* grown in effluent had a lower average in the relative liver weight than the control group on day 14. According to Kalavathy *et al*.^66^, the presence of hepatomegaly may indicate infection, which was not noted in any treatment, showing that *P. pastoris* did not cause adverse effects during the whole period of administration. In addition, although the *Pichia* Effluent group had the highest values of weight gain, no hepatic degeneration or lesions were observed in any of the livers.

In this study, no negative effects of *P. pastoris* were observed on the analyzed parameters, nor any lesion was found in the liver or intestine, regardless of the medium in which the yeast was cultured. These data highlight the innocuous profile of *P. pastoris*, reinforcing previous results in broilers and mice^20–22^. A prior study indicated that the residual biomass of black tea production, composed of *Pichia* sp. NRRL Y-4810 in consortium with two other microorganisms, had beneficial probiotic effects in broilers without causing toxicity or hepatic alterations^70^. In the present study, in the variables in which *P. pastoris* had no superior effect relative to the control, had also no harmful effect, showing that the yeast can be used to increase immunity without impairing the important productive aspects of the quails.

In general, *P. pastoris* grown in effluent supplemented with biodiesel glycerol promoted more benefits in terms of immune status and weight gain, whereas *P. pastoris* grown in YPD improved some egg quality variables. This same trend was observed in broiler chickens, in which *P. pastoris* cultivated in effluent supplemented with biodiesel glycerol induced immunomodulatory effect, while the same yeast grown in YPD improved feed conversion^22^. Our group attributed this to the difference in the composition of the culture medium, which could offer distinct nutrients for the yeast, changing its cellular composition mainly in the cell wall and thereby promoting different interactions with host intestinal mucosa, as has been shown in some studies. As previously suggested, this contrast may be related to the differences in nutrient composition between the culture media^71^. Researchers verified that glycerol may be a carbon source in cultivations of yeasts, enhancing the polysaccharides and mannoprotein contents in the cell wall^72^. They also tested waste potato juice water and glycerol as culture medium for four *S. cerevisiae* strains and detected a modification in the cell wall thickness and changes in the concentrations of mannoproteins and β-glucans^73^. Yeast cell wall mannoproteins act as nonspecific modulators of the immune system and their biological activity may include adjuvant effects^74^ and stimulation of phagocytic activity in macrophages as well as potentiation of synthesis and release of inflammatory mediators as TNF-α and nitric oxide^75^. For these reasons, it is suggested that, due to the diverse composition from the YPD medium, effluent and glycerol can promote differentiated formation of the yeast cell wall, resulting in a unique stimulation of the mucosal immune system. However, analyses of cell wall composition should be performed in the future to prove this effect.

Finally, it is important to note that in most variables, the probiotic effect of *P. pastoris* did not manifest until 84 days, revealing its benefits mainly at the end of experiment. At this time, the more pronounced response to probiotic supplementation probably was attributable to cumulative stress caused by manipulation during the weighing and data collections, long housing period in cages, and decreasing in cage space due to the increase in body size of birds. The poultry sensibility to various stress conditions, such as transport, catching, caging, handling, temperature changes, disturbance, and noise, can affect their performance, cause injury, and even lead to death^76^. The use of probiotics in broiler diets has been shown to alleviate some effects of thermal stress^77,78^ and overcrowding^79^. These studies corroborate what was previously discussed in the present work: the benefits of probiotic supplementation are more evident when the animal is subjected to some type of adverse condition.

In conclusion, *Pichia pastoris* X-33 produced in YPD, when used as a supplement in quail diets caused increased egg weight. When produced in parboiled rice effluent and biodiesel glycerol, *P. pastoris* X-33 had an immunomodulatory effect, enhancing the humoral response to the vaccine against NDV, IBV and Gumboro, without negatively affecting the productive parameters and without causing alterations in the internal organs. The possibility of producing *P. pastoris* X-33 from industrial effluents and by-products makes this yeast an interesting probiotic option for poultry farming.

## Materials and Methods

### *Pichia pastoris* X-33 cultivation

The yeast *P. pastoris* strain X-33 (Invitrogen, USA) was cultured in two distinct culture media: commercial medium Yeast Extract–Peptone–Dextrose (YPD: 1% yeast extract, 2% peptone, 2% dextrose) and an alternative medium composed of parboiled rice effluent + 15 g.L^−1^ of crude glycerol, a by-product of the biodiesel industry, both prepared in laboratory. Commercial culture medium (YPD) was prepared from dilution of the powder YPD medium commercialized by HiMedia (HiMedia ™ Laboratories Pvt Ltd). The alternative medium was prepared using effluent from rice parboiling tanks, provided with no costs by a local rice processing industry, and glycerol provided with no costs by a regional soybean biodiesel industry. After preparation, both media were sterilized. The inoculum was produced in YPD medium at 28 °C for 24 h in an orbital shaker at 180 rpm. Two yeast cultivations were performed in each medium, with 700 mL of inoculum added to 6.3 L of medium in a bench bioreactor (Bioflo 110, New Brunswick) at 28 °C, 500 rpm, 1 vvm of air, for 24 hours. The pH was adjusted to 5.5 with 1 M NaOH, and antifoam (Antifoam 204, Sigma) was added to the cultures to avoid excessive foaming as a result of stirring. After cultivation, cells were recovered by centrifugation at 4,000 rpm for 15 minutes and then washed three times with 0.9% saline solution. Viable cells of *P. pastoris* X-33 were quantified by plating serial dilutions in YM Agar and counting of colony forming units (CFU.mL^−1^) after incubation at 28 °C for 48 h.

### Animals and housing conditions

In total, 106 forty-days-old female quails, from a *Coturnix coturnix coturnix* lineage, were used in the experiment. The animals belonged to a dual purpose lineage developed in Federal University of Pelotas, which has good performance on both egg productivity and meat production. The quails were housed in metal cages, in a room with controlled temperature around 25 °C and cycles of 17 h of light and 7 h of darkness. During the experimental period, the birds received water *ad libitum* and the feed was provided daily.

### Ethical approval statement

The procedures and activities performed in this experiment were approved by Federal University of Pelotas Committee on Animal Research and Ethics, protocol n° 6848, in agreement with the Brazilian legislation, relevant guidelines and regulations, following all the ethical precepts of animal experimentation.

### Experimental design

The experiment lasted for 84 days, divided into three consecutive cycles of 28 days each. Birds were previously weighed and distributed among the treatments according to their weights, in order to maintain the homogeneity among treatments above 80%.

### Treatments

The birds were divided into three treatments, with 16 repetitions each: T1 (Control): Vaccinated animals that received basal diet without yeast; T2 (*Pichia* Effluent): Vaccinated animals receiving a basal diet supplemented with *Pichia pastoris* produced in parboiled rice effluent supplemented with biodiesel glycerol, and T3 (*Pichia* YPD): Vaccinated animals receiving a basal diet supplemented with *Pichia pastoris* produced in YPD medium. The repetitions consisted of an experimental unit of two quails, and each repetition was allocated in an individual metal cage. A separated group of 10 animals did not receive the vaccines and served as a control for immunomodulation assays. These animals received basal diet without yeast and were housed under the same conditions as the treatment groups throughout the experimental period.

### Experimental diets

A basal diet without antimicrobials was formulated specifically to meet the nutritional needs of the quails, according to the recommendations^80^, as shown in Table 8. The diets were isocaloric, isoproteic and isovitaminic, differing only by the addition “on top” of *P. pastoris* X-33, in the concentration of 1 × 10^7^ CFU.g^−1^, in T2 and T3. Yeast was mixed into the diet weekly, followed by quantification to assess cell viability and confirm the appropriate concentration. After preparation, the diets were stored in individual containers for each repetition, so that feed consumption per repetition could be better controlled.

**Table 8:** Composition of basal diet.

Ingredient	Content (%)
Corn, grain	44.60
Soybean meal	40.38
Limestone	5.83
Vitamin mineral mix^b^	5.00
Soybean oil	2.50
Dicalcium Phosphate	1.20
DL-Methionine	0.22
Sodium chloride	0.18
L-Lysine HCl	0.08
**Nutrient Composition**	
ME (kcal/kg)^a^	2805
CP (%)^a^	21.98
Digestible lysine (%)	1.18
Digestible methionine (%)	0.50
Digestible methionine + cysteine (%)	0.80
Available phosphorus (%)	0.34
Calcium (%)	3.50
Sodium (%)	0.24
Chloride (5)	0.15
Threonine (%)	0.75
Tryptophan (%)	0.26

^a^ME = metabolizable energy; CP = crude protein.

^b^Composition per kilogram of product: zinc: 1535 mg; manganese: 1485 mg; iron: 1695 mg; iodine: 29 mg; copper: 244 mg; selenium: 3,2 mg; calcium: 197,5 g; cobalt: 5,1 mg; fluoride: 400 mg; phosphorus: 50 g; methionine: 11 g; vitamin E: 540 mg; vitamin B1: 40 mg; vitamin B6: 54 mg; vitamin K3: 51,5 mg; vitamin B12: 430 mcg; vitamin A: 207,000 IU; vitamin D3: 43,200 IU; vitamin B2: 120 mg; pantothenic acid: 204,6 mg; choline: 320 mg; biotin: 1,4 mg; folic acid: 16,7 mg; nicotinic acid: 840 mg.

### Vaccination

On day one of the experiment, the birds were vaccinated with the New-Bronk-Gumbor (Biovet®) vaccine against Newcastle Disease, Infectious Bronchitis, and Gumboro Disease. In each bird was administered a dose of 0.25 mL intramuscularly in the chest muscle. After 28 days, the birds were revaccinated with the same dose.

### Performance

The following performance parameters were evaluated: weight gain, feed intake, egg production, and feed conversion. The birds were weighed at the beginning of the experimental period and at the end of each cycle. Egg weight was determined weekly and egg laying of each animal was noted daily to calculate the egg production (P) according to the formula: P (%) = (total number of eggs produced in the cycle × 100)/number of days of the cycle. The feed intake in each experimental unit was determined weekly by the difference between the amount of diet provided for seven days and the leftover at the end of this period. The egg mass was calculated by the formula: Egg mass = (Average egg weight × egg production)/100. For egg weight, two eggs of each experimental unit were weighed once a week, and from these values, the week average of egg weight of the treatments was calculated and then the average for each 28-day cycle was performed from the week values. Feed conversion per egg mass was defined by the ratio between feed intake and egg mass (g.g^−1^).

### Egg quality

In the last three days of each cycle, eggs were collected for external and internal quality analysis. Two eggs per experimental unit were chosen randomly to be used in the analyses.

#### External quality

The eggs were weighed individually on a digital scale with a precision of 0.01 g. To measure the specific gravity, a variable related to egg shell quality, the eggs were placed in successive containers with solutions of increasing concentrations of sodium chloride (NaCl) until they floated. The density at which the flotation occurred was noted for each egg^81^. The eggs were then broken, and the shells were washed, dried at room temperature for 72 hours, and weighed individually on an analytical digital scale with a precision of 0.0001 g. The eggshell percentage was calculated as the eggshell weight multiplied by 100 and divided by the egg weight. The eggshell thickness was measured in the equatorial region of the egg using a digital micrometer (Digimess®) with a precision of 0.001 mm^82^.

#### Internal quality

The opened eggs were placed individually in Petri dishes. Yolk color was evaluated visually with a yolk color fan (DSM^®^) containing a 15-tone color scale ranging from 1 (light yellow) to 15 (orange-red). Yolk coloration was also analyzed using a portable digital colorimeter (Minolta®) according to the methodology proposed by Honikel^83^ for quantification of the parameters: L* (lightness), a*, and b*. The a* value involves the color component that varies from red (+a*) to green (−a *), the b* value varies from yellow (+b*) to blue (−b*), and the L* value varies from white (L = 100) to black (L = 0).

The Haugh unit was obtained from the formula described by Haugh^84^: UH = 100 × log (H + 7,57 − 1,7 × W^0,37^), where H = albumen height and W = egg weight. For this, the albumen height was measured with a manual pachymeter. The yolk and albumen weights were determined with a precision digital scale, and then multiplied by 100 and divided by the egg weight to determine the yolk and albumen percentages, respectively.

### Intestinal morphometry, organ weights and safety

Four animals from each treatment were slaughtered on days 14, 28, 56, and 84 of the experiment. After 24 hours of fasting, the quails were euthanized, eviscerated, and the heart, liver, spleen, bursa of Fabricius, cecal tonsils, and duodenum were collected whole and weighed on an analytical digital scale. Liver portions and 2 cm sections of the duodenum were fixed in a 10% formalin solution for 48 h and subsequently dehydrated by successive washes with ethyl alcohol in increasing concentrations (70%, 80%, 90%, and absolute). After dehydration, the samples were diaphanized in xylol, followed by inclusion in paraffin blocks, which were cut with a microtome in 12 transverse and semi-seriated cuts with a thickness of 5 μm. The blades were stained with hematoxylin-eosin and covered with coverslips. The livers were analyzed for the presence of lesions, while the morphometry of duodenums was evaluated using image capture and measurement of villus height and crypt depth with Image Pro-Plus 4.5 software (Media Cybernetics, Silver Spring, MD).

### Humoral immune response

Blood samples were collected from animals to be slaughtered on days 14, 28, 56 and 84 to quantify antibodies to the New-Bronk-Gumbor (Biovet®) vaccine by indirect ELISA. Blood samples from non-vaccinated animals were also collected and tested, as a control of the assay. Monovalent vaccines against Newcastle disease (New-Vacin La Sota), Infectious Bronchitis (Bio-Bronk-Vet H-120), and Gumboro disease (Gumbor-Vet) were used as antigen. All the 96-well microtiter plates were coated with two doses of vaccine per well, resuspended in carbonate-bicarbonate buffer (pH 9.6), and incubated overnight at 4 °C. After washing with PBS-T, sera were diluted 1:50, added in triplicate, and incubated for 1 h at 37 °C. Specific antibodies were detected with the peroxidase-conjugated anti-chicken secondary antibody, diluted 1:2000. After washing with PBS-T, chromogenic substrate was added, and the absorbances were quantified with spectrophotometer at 450 nm.

### Hemagglutination inhibition test

The antibody titer against Newcastle disease virus (NDV) was determined from the sera of days 14, 28, 56, and 84 by the hemagglutination inhibition (HI) test, according to the standard protocol^85^. Sera of the non-vaccinated animals were also tested, as a control of the assay. Initially, a hemagglutination test was performed to determine the dilution corresponding to four hemagglutinating units (4 HAU) of the virus. One HAU is considered the reciprocal of the highest dilution in which occurred complete agglutination of a 1% suspension of chicken erythrocytes. For the HI test, 25 μL of sera were added to microplates containing 25 μL/well of PBS for base-2 serial dilutions, up to 1:4096. Four HAUs of the virus were loaded into each well, and after 30 minutes of incubation, 25 μL of a 1% suspension of chicken erythrocytes were added to the wells. The plate was incubated at rest at room temperature for 30 minutes to allow the sedimentation of the erythrocytes. The titer of each serum was expressed as the log_2_ of the reciprocal of the highest serum dilution which inhibited the hemagglutinating activity of NDV.

### Statistical analysis

To verify the effects of yeast and quail age on performance and egg quality variables, statistical analysis was performed according to a completely randomized design with time-repeated measures using the Mixed procedure and Compound Symmetry covariance matrix structure. After analysis of variance (ANOVA), the LSM (Least Squares Means) test was performed, with the comparison of means adjusted by the Tukey-Kramer test. In the results of intestinal morphometry and antibody quantification, the Statistix 9 software was used to perform ANOVA and Tukey test to determine the significance levels between the means of the treatments. All statistical tests were performed with a significance level of P < 0.05.

## Data Availability

The authors declare that materials, data and associated protocols are promptly available to readers without restrictions. Readers can obtain materials and information under request, contacting the corresponding author.
